# Interneuron Progenitor Transplantation to Treat CNS Dysfunction

**DOI:** 10.3389/fncir.2016.00064

**Published:** 2016-08-17

**Authors:** Muhammad O. Chohan, Holly Moore

**Affiliations:** ^1^Department of Integrative Neuroscience, New York State Psychiatric Institute, New YorkNY, USA; ^2^Department of Psychiatry, Columbia University, New YorkNY, USA

**Keywords:** stem cell therapy, GABA, medial ganglionic eminence, excitation-inhibition, neuropsychiatric disorders

## Abstract

Due to the inadequacy of endogenous repair mechanisms diseases of the nervous system remain a major challenge to scientists and clinicians. Stem cell based therapy is an exciting and viable strategy that has been shown to ameliorate or even reverse symptoms of CNS dysfunction in preclinical animal models. Of particular importance has been the use of GABAergic interneuron progenitors as a therapeutic strategy. Born in the neurogenic niches of the ventral telencephalon, interneuron progenitors retain their unique capacity to disperse, integrate and induce plasticity in adult host circuitries following transplantation. Here we discuss the potential of interneuron based transplantation strategies as it relates to CNS disease therapeutics. We also discuss mechanisms underlying their therapeutic efficacy and some of the challenges that face the field.

## Introduction

Since the turn of 20th century neural transplantation has been studied as a potential therapeutic strategy for neural reconstruction and repair (Cajal, 1928). Initial attempts with engraftment of neural tissue in lower vertebrates remained largely unsuccessful with most studies concluding that the adult brain was inhospitable for graft survival (Willis, 1935). However, several early studies showed promise with engraftment of developing tissue (Dunn, 1917; Tidd, 1932; Clark, 1940). Notably, one study (Clark, 1940) demonstrated viability of embryonic neocortical grafts dissected from 15 to 20 days old embryos transplanted in the young mammalian brain. Strikingly, the engrafted cells differentiated and migrated such that the graft showed laminar features and cell morphologies characteristic of the developing cerebral cortex. Later, transplanted neonatal and embryonic cells were shown to develop normal synaptic afferent and efferent connections with host brain and spinal neurons (Björklund et al., 1976; Lund and Hauschka, 1976; Jakeman and Reier, 1991). Moreover, differentiation and integration of transplanted cells within the adult striatum and hippocampus was shown to be associated with improvements in motor coordination and spatial learning (Low et al., 1982; Gage et al., 1983, 1984; Björklund and Stenevi, 1984). Later studies revealed details of the anatomical and functional integration of the grafted tissue into host circuits: neurons within intraspinal grafts that were effective in improving motor function were shown to extend axons along white matter tracts rostrally and caudally (Nornes et al., 1983; Jakeman and Reier, 1991; Li and Raisman, 1993), and form synaptic relays (Cummings et al., 2005; Lu et al., 2012). Together, these studies revealed wide ranging potential for neural stem cell transplantation in CNS disease therapeutics.

## Factors Affecting Viability and Functionality of A Neuronal Transplant

In several studies the ideal age of embryonic donor tissue has been shown to depend on the developmental stage of the donor tissue, the time window including neuronal proliferation and migration being optimal (Banker and Cowan, 1977; Kromer et al., 1983). This is consistent with the finding that isolated hippocampal neurons that have recently completed DNA synthesis and are in the process of migration showed long term survival in cultures as opposed to post-mitotic cells dissociated from the ventricular zone or the cortical plate (Banker and Cowan, 1977). Other favorable attributes of young tissue may include their relative insusceptibility to transplant procedure related trauma, as well as their particularly low 0_2_ consumption rate which may buoy their viability in new environments during the initial days after implantation (Shyh-Chang et al., 2013).

A major objective of transplant studies has been to determine factors that allow the grafted cells to integrate functionally into host circuits, as such integration is thought to be important for sustainable therapeutic effects. In early studies it was noted that some types of transplanted embryonic cells displayed minimal dispersion, with functional effects often limited to graft site. For example, while cells within embryonic nigral tissue grafted into the striatum of 6-OHDA lesioned rats differentiate into dopamine neurons, the neurons do not disperse, but rather form a dopamine “island” near the site of transplantation within the striatum. Considering these observations, major factors contributing to a transplant’s long term functionality would be the age of donor tissue, its ability to demonstrate widespread dispersion and integration within host, as well as the use of disease relevant cell types.

## Interneuron Progenitors As A Candidate For Cell Based Therapy

**FIGURE 1:**
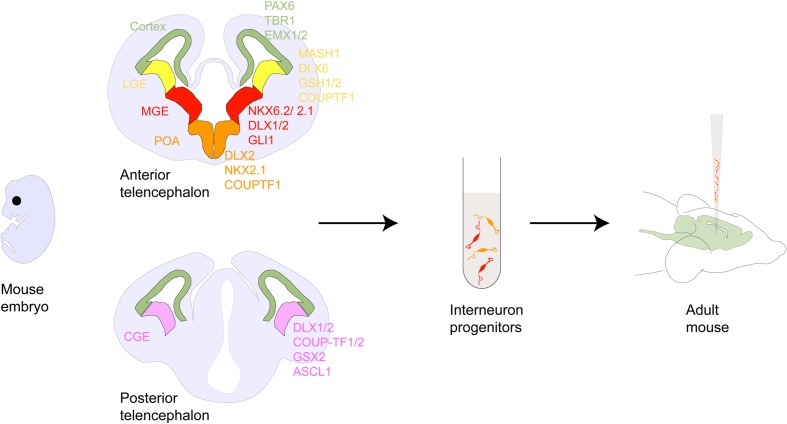
**Interneuron development within the forebrain.** Schematic showing coronal sections of the rodent anterior and posterior telencephalon. Shown are the pallial and subpallial neurogeneic niches and their gene expression patterns. Following the discovery of tangentially migrating neurons in the developing cortex (Walsh and Cepko, 1988, 1993) several cell lineage studies demonstrated that many of these neurons originated in the ventral telencephalic ganglionic eminences and the POA and ultimately matured into GABAergic interneurons. For example, ablation of the embryonic ventral telecephalon or mutation of the homeodomain transcription factor gene Dlx1/2 heavily expressed in this region results in a dramatic loss of neocortical GABAergic neuron populations (Anderson et al., 1997). Ventral fate inducing sonic hedgehog(SHH) signaling has been shown to specify PV and SST expressing interneuron differentiation from ventral telencephalic progenitors in culture and *in vivo* (Xu et al., 2010; Tyson et al., 2015). Several other homeobox genes such as Mash1 (Casarosa et al., 1999), Nkx2.1 (Sussel et al., 1999), Lhx6 (Liodis et al., 2007), and FGFR (Gutin et al., 2006), as well as guidance cues such as the chemorepulsive semaphorin/neuropilin (Marín et al., 2001) and chemoattractive neuregulin-1/ErbB4 (Flames et al., 2004) interactions have been shown to be critical for proper direction and selection of GABAergic cell identity, subtype and migration. Genetic fate mapping of transplanted embryonic cells in cultures or *in utero* has revealed that MGE gives rise to PV- and SST-, while CGE gives rise to CR-, VIP- and Reelin-expressing interneurons, with each cell group having a distinct spatio-temporal origin (Nery et al., 2002; Xu et al., 2004; Butt et al., 2005; Miyoshi et al., 2010; Petros et al., 2015). In addition, the POA has been shown to be the origin of at least 10 percent of GABAergic interneurons comprising of PV-, SST- and a small percentage of VIP-, NOS-, and CR-expressing interneurons (Gelman et al., 2011). Abbreviations: LGE, MGE, CGE (lateral, medial, caudal ganglionic eminence); POA (preoptic area); CB (cabindin), CR (calretinin), PV (parvalbumin), SST (somatostatin), NPY (neuropeptide Y), RLN (reelin), NOS (nitrous oxide) expressing interneurons.

While the early studies served as proof-of-principle for the concept of cell based therapy, mechanisms by which transplanted cells modify diseased brain circuitries have remained largely unknown. With refined knowledge of neurodevelopment (Merkle and Alvarez-Buylla, 2006; Muotri and Gage, 2006; Fuentealba et al., 2015) studies carried out over the past two decades have now begun to offer mechanistic insights and renewed evidence for the therapeutic efficacy of cell transplantation in CNS diseases. Of particular relevance has been the use of γ-amino butyric acid (GABA)-ergic inhibitory neuron precursors (Wonders and Anderson, 2006; Tricoire et al., 2011; Southwell et al., 2014) (**Figure 1**). Constituting only about 20% of the adult cortical neuronal population, inhibitory neurons are potent regulators of normal brain function, sculpting the excitation-inhibition balance and entraining activity of neuron ensembles in brain circuits (Klausberger et al., 2003; Klausberger and Somogyi, 2008; Lewis et al., 2012). Maturation of GABA circuits has been shown to set off and regulate critical period plasticity in brain sensory systems, offering a putative neurobiological handle with which to interrogate neurodevelopmental origins of neurological disorders (Hensch, 2005). As such imbalances in excitation-inhibition and dysfunction of inhibitory interneurons are hypothesized to underlie several neurological disorders like schizophrenia (SCZ; Harrison, 2015), autism spectrum disorders (Peñagarikano et al., 2011), Alzheimer’s disease (AD; Andrews-Zwilling et al., 2010), Parkinson’s disease (PD; Salin et al., 2009), epilepsy (Möhler et al., 2004), and neuropathic pain (NP) (Moore et al., 2002).

## Epilepsy

Impaired inhibition has been described as a key pathognomonic feature in animal models of (Sloviter, 1987; Cossart et al., 2001) and human patients with (de Lanerolle et al., 1989; Mathern et al., 1995) epilepsy. For example, studies in temporal lobe epilepsy, the most common type in adults, have revealed deficits in hippocampal pyramidal neuron distal dendritic domain-targeting interneurons, including interneuron subpopulations expressing somatostatin (SST; Buckmaster and Jongen-Rêlo, 1999; Cossart et al., 2001; Kobayashi and Buckmaster, 2003), neuropeptide Y (NPY; Mathern et al., 1995; Sundstrom et al., 2001), and calbindin (CB; Wittner et al., 2002). In addition, a decrease in the density of hippocampal basket and chandelier parvalbumin (PV) immunoreactive cells has been reported (DeFelipe, 1999; Arellano et al., 2004; Ogiwara et al., 2007); however, perisomatic GABA innervation appears to be intact (Wittner et al., 2001, 2005). Despite compensatory sprouting of interneurons (Mathern et al., 1995; Arellano et al., 2004) imbalances in input-output relationship of pyramidal cells (Cossart et al., 2001) result in abnormal cortical network activity (Ogiwara et al., 2007). Introducing functional GABAergic neurons hence provides a means of replacing lost or dysfunctional inhibitory cells and tempering increased electrical activity seen in epilepsy.

**FIGURE 2:**
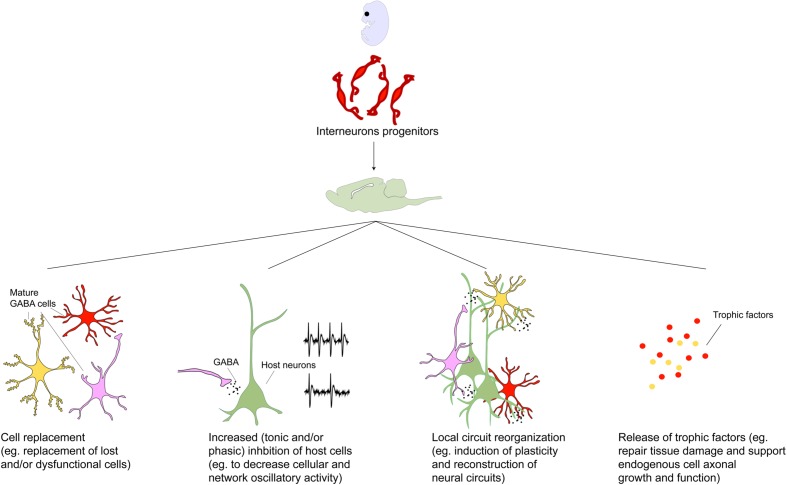
**Toward a mechanistic understanding of transplant induced therapeutics.** Transplanted GABAergic interneurons make synaptic connections and enhance inhibitory tone within the host brain region (Alvarez-Dolado et al., 2006; Baraban et al., 2009). The importance of these new synaptic connections has been demonstrated in the mature visual cortex where interneuron progenitor implantation but not pharmacological GABA augmentation induces cortical plasticity and reopening of critical period (Fagiolini and Hensch, 2000; Hensch, 2005; Southwell et al., 2010; Davis et al., 2015). This has been attributed to the numerous, individually weak inhibitory connections made by the transplanted neurons which may trigger local circuit reorganization (Southwell et al., 2010). However, given that the behavioral effects of GABAergic interneuron transplants are not phenocopied by local pharmacological enhancement of GABA transmission, and sparseness of transplanted neurons relative to the host population, it has been hypothesized that therapeutic effects of transplants may be mediated by mechanisms beyond that of GABA replacement (Gilani et al., 2014; Southwell et al., 2014). Intriguingly, transplanted neural stem cells have been demonstrated to rescue dysfunctional host dopamine neurons in MPTP-treated aged animals (Ourednik et al., 2002). Other proposed effects independent of synaptic connections with host neurons include secretion of neuroprotective factors (Capone et al., 2007; Redmond et al., 2007; Liang et al., 2014; Goldberg et al., 2015). In addition transplanted neural cells have been proposed to create homeostatic environments by releasing factors that repair and counter inflammation and scarring (Park et al., 2002; Lee et al., 2007). More recently, interrogation of host circuit physiology following engraftment of undifferentiated and non-integrating cells revealed a dose-dependent dampening of cortical excitability and depletion of host cell populations underscoring the importance of engraftment density in designing cell based therapeutic protocols (Weerakkody et al., 2013). Considering the wide-ranging repertoire of transplant-induced effects, discovery of molecular mechanisms underlying these may uncover novel treatment targets in the future.

Many of the early interneuron progenitor transplantation studies were carried out in animal models of epilepsy. Alvarez-Dolado et al. (2006) provided the first electrophysiological evidence of functional integration of MGE derived neuronal precursors grafted into the juvenile brain. In this study, grafted MGE cells within the hippocampus migrated up to ∼5 mm from injection site at 2 months after transplantation, acquired molecular markers of mature GABAergic interneurons [by expressing GABA, PV, SST, calretinin (CR), and NPY] and increased GABA mediated synaptic inhibition in regions containing transplants. In a series of follow-up studies, therapeutic efficacy of MGE progenitors was demonstrated by the reduction of severity and frequency of seizures in genetic (Baraban et al., 2009; Hammad et al., 2015) and acquired (Hunt et al., 2013; Henderson et al., 2014; Jaiswal et al., 2015) rodent models of epilepsy and hippocampal disinhibition (Calcagnotto et al., 2010; Waldau et al., 2010). Notably, while MGE cell transplantation in the hippocampus increased inhibitory post-synaptic current (IPSC) frequencies in host pyramidal (Baraban et al., 2009) and granule cells (Henderson et al., 2014), it did not significantly alter IPSC properties of host interneurons (Baraban et al., 2009), whose inhibition is mediated by interneuron subclasses generated from the caudally located CGE cells (Gulyás et al., 1996; Wonders and Anderson, 2006; Caputi et al., 2009). Remarkably, therapeutic efficacy was demonstrated as early as 2.5 weeks following transplantation suggesting possible roles for non-synaptic mechanisms in disease amelioration (**Figure 2**; De la Cruz et al., 2011). Translational significance of these findings has been tested using transplantation of human pluripotent stem cell derived MGE cells in a pilocarpine-induced temporal lobe epilepsy mouse model (Cunningham et al., 2014; Hunt and Baraban, 2015). While MGE transplants have been shown to increase both synaptic and extrasynaptic inhibition onto host pyramidal neurons (Baraban et al., 2009), activation of extrasynaptic GABA receptors was reported as the basis of MGE precursor transplantation induced amelioration of seizure activity in the cortex (Jaiswal et al., 2015). Given the differential involvement of heterogeneous interneuron populations in the compensatory and epileptogenic mechanisms, including disease initiation and exacerbation (Cohen et al., 2002; Panuccio et al., 2009), GABA interneuron transplants may prove therapeutic for some types or stages of epilepsy. Key to the success of transplants will be to identify which types or stages of epilepsy can benefit and determine the effective composition of the transplant. This will be aided by rapidly developing technologies that allow high throughput generation of developmentally and functionally distinct interneuron subclasses (Doudna and Charpentier, 2014; Colasante et al., 2015).

## Schizophrenia and Cortical Plasticity

Reductions in SST and PV expressing neurons or functional markers thereof have been observed in the hippocampus and prefrontal cortex in postmortem brains of patients with SCZ (Hashimoto et al., 2003, 2008; Morris et al., 2008; Konradi et al., 2011). Indeed, disturbances in synchronous cortical oscillations, generated by fast spiking PV neurons (Sohal et al., 2009) and that are correlated with cognitive functions, are a physiological feature of SCZ (Murray et al., 2011). Moreover, increased hippocampal blood volume, a proxy for metabolic activity, a reliable feature of SCZ and correlate of psychosis, can be produced by functional deficits in GABAergic interneurons (Schobel et al., 2013; Gilani et al., 2014).

Exploring the role of cortical interneurons in schizophreniform cognitive deficits in mice, Tanaka et al. (2011) demonstrated that transplantation of MGE cells in the cortex can prevent phencyclidine induced behavioral deficits, possibly via modulation of local cortical circuitry (Southwell et al., 2010; Howard et al., 2014). Moreover, in a genetic mouse model displaying a relatively selective deficit in hippocampal PV interneurons (Glickstein et al., 2007), transplantation of MGE cells into the hippocampus reversed increased hippocampal activity (as measured with functional magnetic resonance imaging), increased midbrain dopamine neuron activity, increased response to psychostimulants and impaired hippocampus dependent cognition (Gilani et al., 2014). Given the central role of GABA in controlling the timing of critical period plasticity (Hensch, 2005) MGE cells were transplanted in the visual cortex to test whether transplantation of inhibitory cells could induce a plasticity response (Tang et al., 2014). Remarkably, MGE cell transplantation either before (Southwell et al., 2010) or after (Davis et al., 2015) the endogenous critical period induced ocular dominance plasticity and reactivated a new critical period that reversed visual impairments in amblyopic mice. Importantly both PV and SST neuronal populations were shown to be sufficient to drive ocular dominance plasticity but grafts depleted of both PV and SST populations were not (Tang et al., 2014). Intriguingly, maturing GABA neurons formed numerous but individually weak synaptic connections with host neurons suggesting a potential reorganization of local cortical circuitry. Sufficiency of both PV and SST interneuron populations to induce plasticity in the visual cortex raises an interesting question as to whether the same also holds true for other brain regions and the spinal cord, as it may have implications for developing refined cell based therapies.

## Alzheimer’s Disease

GABAergic control of synaptic plasticity is a key aspect of hippocampus dependent learning as it relates to encoding and retrieval of memories (Paulsen and Moser, 1998). AD patients have been shown to have decreased GABA and SST immunoreactivity in the cerebral cortex (Davies et al., 1980; Grouselle et al., 1998), and in late-onset familial and sporadic AD, the risk gene apolipoprotein (apo) E4 polymorphism exacerbates SST dysfunction (Grouselle et al., 1998). In addition, misfolded proteins such as amyloid-β that are seen in early-onset autosomal dominant AD cause inhibitory interneuron impairments resulting in excitation-inhibition imbalances and network hyperexcitability that is in turn associated with learning and memory deficits (Palop et al., 2007; Andrews-Zwilling et al., 2010; Verret et al., 2012). In particular, PV cell dysfunction has been causally linked to disturbances in cortical network oscillations and cognitive impairments in mice expressing human amyloid precursor protein (Verret et al., 2012). Indeed, AD patients are known to have increased incidence of epileptic events (Amatniek et al., 2006).

In septo-hippocampal lesioned mice, intrahippocampal transplantation of subventricular zone-expanded neural stem cells (Hattiangady and Shetty, 2012) or human embryonic stem cell induced MGE cells (Liu et al., 2013) were shown to reverse learning and memory deficits. Importantly, control experiments involving transplants of spinal progenitors failed to correct these deficits, highlighting the requirement of specific neuronal progenitors in a given target region to achieve functional replacement (Liu et al., 2013). These findings were later extended in two genetic mouse models of AD: apoE4 knock-in mice with or without amyloid-β protein accumulation. Importantly, MGE derived cells showed functional integration in the presence of amyloid aggregation and restored cognitive function thus demonstrating interneuron progenitors’ ability to integrate and modify diseased circuits in toxic environments (Tong et al., 2014). Given the strong evidence of impaired synaptic inhibition (Busche et al., 2008) and network oscillatory activity (Amatniek et al., 2006) in AD, GABA cell replacement strategies especially those employing fast spiking PV neurons could be a promising therapeutic avenue.

## Parkinson’s Disease

Grafts of dopaminergic cell suspensions into 6-hydroxydopamine (6-OHDA) lesioned striatum are known to restore motor deficits (Gage et al., 1983). However, a critical limitation of dopaminergic cell grafts is the inability of transplanted cells to disperse in host tissue, limiting the functional recovery to graft site. Intriguingly, local striatal GABAergic interneuron population activity has been shown to sculpt basal ganglia output (Tepper and Bolam, 2004) and abnormalities in inhibitory neurons underlie striatal output imbalances in the dopamine depleted striatum (Mallet et al., 2006). Striatal interneurons may also regulate basal ganglia plasticity (Cazorla et al., 2014) by modulating striatal extrinsic and intrinsic signals and thus may serve as a potential non-dopamine locus to lower striatal activity characteristic of PD.

Neurons derived from transplants of MGE tissue in striatum of the 6-OHDA lesioned mice migrate, functionally integrate and improve motor deficits (Martínez-Cerdeño et al., 2010). Under dopamine depleted conditions, striatopallidal neuronal activity is increased while the opposite is true for striatonigral neurons (Shen et al., 2008). Moreover, selective increase in feed-forward inhibition from local PV interneurons onto striatopallidal neurons enhances neural synchrony (Gittis et al., 2011) which may lead to aberrant β-oscillatory activity characteristic of PD. Overall, GABA tone is increased in the basal ganglia (Borgkvist et al., 2015) leading to persistent suppression of action. Since the transplanted cells show synaptic integration, one possible mechanism of action underlying therapeutic efficacy of MGE cells is the restoration of the balance between the direct and indirect pathways. However, it is notable that a large proportion of donor cells differentiate into oligodendrocytes, implicating non-neuronal support functions provided by the grafts (**Figure 2**). Intriguingly, MGE transplants made in the subthalamus fail to migrate from the injection site and instead differentiate into glial cells that show long term survival, highlighting the critical role donor-host environmental interactions may play in governing fate of transplanted progenitor cells.

## Neuropathic Pain

In contrast to cortex, inhibitory interneurons form about 30–40% of the total neuronal population in the dorsal horn of the spinal cord. By regulating activity of primary afferents, excitatory interneurons, spinal projection neurons and descending fiber tracts, inhibitory interneurons play a crucial role in maintaining a physiological level of pain sensitivity. Disinhibition within the spinal dorsal horn has long been attributed to the symptoms of NP, e.g., GABA neurotransmission is significantly reduced following nerve injury (Moore et al., 2002; Drew et al., 2004) and GABA agonists can ameliorate allodynia and hyperalgesia (Munro et al., 2009). Impaired inhibition, however, results in multiple cellular, molecular and synaptic changes and therapeutic efficacy of currently available anticonvulsant- and antidepressant-based pharmacological agents is at best symptomatic and constrained by the drugs’ broad mechanisms of actions. Given the ability of interneuron progenitors to functionally integrate in neural tissue and modify inhibitory signaling, interneuron transplantation could serve as a potential disease modifying strategy in NP that is without the adverse side effects associated with systemic medications.

Surprisingly, transplanted MGE cells into adult spinal cord differentiate into GABA interneuron populations that integrate with the host spinal cord circuitry (Bráz et al., 2012). Contrary to subthalamic MGE grafts that differentiate into glial cells (Martínez-Cerdeño et al., 2010), MGE derived cells in the spinal cord retain their cortical neurochemical profiles suggesting that the latter does not affect MGE differentiation. More importantly, MGE transplantation reversed mechanical hypersensitivity in a mouse model of peripheral nerve injury (Bráz et al., 2012) and mechanical and heat hyperalgesia in a chemotherapy-induced model of NP (Bráz et al., 2015). Notably, transplantation of cells lacking vesicular GABA transporter failed to rescue paclitaxel-induced pain behavior highlighting the critical role of GABA-mediated modulation of spinal circuits in transplant efficacy in this pain model (Bráz et al., 2015). Given the problems with tolerance and the sedative and addictive properties of traditional pharmacotherapies, interneuron based therapy may be a promising alternative disease modifying therapeutic option.

## Challenges and Pitfalls in Using MGE Progenitor Cell Based Therapy

While pre-clinical studies have demonstrated promise for interneuron based therapy, cell based approaches have inherent limitations, including but not limited to unpredictable proliferation, differentiation, and migration, leading to pathological ectopia, including tumors. Addressing these limitations will require greater understanding of the mechanisms governing the development and function of the transplants in different brain regions and at different points in the lifespan of the host (**Figure 2**). Combined with these challenges, incomplete knowledge of the pathophysiology of vast majority of CNS diseases hinders progress to clinical translation. While excitement surrounds generation of GABAergic interneurons from human pluripotent stem cells (Maroof et al., 2013; Wall et al., 2013), current techniques have yet to achieve sufficient efficiency and specificity. Technological innovations are leading to introduction of genetic material into transplanted cells to increase specificity, improvement in production methods allowing more rapid amplification, differentiation of stem cells, and use of cell sorting techniques to select cells on the basis of what will comprise a safe transplant designed specifically to improve function in a given brain region. Identifying host characteristics that can aid survival and guide maturation of the grafted cells *in vivo* will also address these challenges.

## Conclusion

The remarkable capacities of interneuron progenitors to migrate long distances, differentiate into mature interneurons and modify diseased circuits following transplantation have made interneuron based transplantation a viable potential therapeutic approach for CNS diseases. However, further studies in the primate, a refined knowledge of interneuron ontogenesis and development of methods for reliable, high-throughput production of specific GABAergic cell types and safe cell composition of transplants need to be pursued before this approach is realized in the clinical setting. There is reason to be optimistic, given rich and growing literature on interneuron development and rapid growth of technologies that will allow the production of safe and specific transplants.

## Author Contributions

MC and HM developed concepts. MC wrote and HM edited the paper.

## Conflict of Interest Statement

The authors declare that the research was conducted in the absence of any commercial or financial relationships that could be construed as a potential conflict of interest.

## References

[B1] Alvarez-Dolado M. Calcagnotto M. E. Karkar K. M. Southwell D. G. Jones-Davis D. M. Estrada R. C. (2006). Cortical inhibition modified by embryonic neural precursors grafted into the postnatal brain. *J. Neurosci.* 26 7380–7389. 10.1523/JNEUROSCI.1540-06.2006 16837585 PMC1550786

[B2] Amatniek J. C. Hauser W. A. DelCastillo-Castaneda C. Jacobs D. M. Marder K. Bell K. (2006). Incidence and predictors of seizures in patients with Alzheimer’s disease. *Epilepsia* 47 867–872. 10.1111/j.1528-1167.2006.00554.x 16686651

[B3] Anderson S. A. Eisenstat D. D. Shi L. Rubenstein J. L. (1997). Interneuron migration from basal forebrain to neocortex: dependence on dlx genes. *Science* 278 474–476. 10.1126/science.278.5337.474 9334308

[B4] Andrews-Zwilling Y. Bien-Ly N. Xu Q. Li G. Bernardo A. Daniel Zwilling S. Y. (2010). Apolipoprotein E4 causes age- and tau-dependent impairment of GABAergic interneurons, leading to learning and memory deficits in mice. *J. Neurosci.* 30 13707–13717. 10.1523/JNEUROSCI.4040-10.2010 20943911 PMC2988475

[B5] Arellano J. I. Muñoz A. Ballesteros-Yáñez I. Sola R. G. DeFelipe J. (2004). Histopathology and reorganization of chandelier cells in the human epileptic sclerotic hippocampus. *Brain* 127 45–64. 10.1093/brain/awh004 14534159

[B6] Banker G. A. Cowan W. M. (1977). Rat hippocampal neurons in dispersed cell culture. *Brain Res.* 126 397–425. 10.1016/0006-8993(77)90594-7 861729

[B7] Baraban S. C. Southwell D. G. Estrada R. C. Jones D. L. Sebe J. Y. Alfaro-Cervello C. (2009). Reduction of seizures by transplantation of cortical GABAergic interneuron precursors into Kv1.1 mutant mice. *Proc. Natl. Acad. Sci. U.S.A.* 106 15472–15477. 10.1073/pnas.0900141106 19706400 PMC2741275

[B8] Björklund A. Stenevi U. (1984). Intracerebral neural implants: neuronal replacement and reconstruction of damaged circuitries. *Annu. Rev. Neurosci.* 7 279–308. 10.1146/annurev.ne.07.030184.001431 6370080

[B9] Björklund A. Stenevi U. Svendgaard N. (1976). Growth of transplanted monoaminergic neurones into the adult hippocampus along the perforant path. *Nature* 262 787–790. 10.1038/262787a0 958453

[B10] Borgkvist A. Avegno E. M. Wong M. Y. Kheirbek M. A. Sonders M. S. Hen R. (2015). Loss of striatonigral GABAergic presynaptic inhibition enables motor sensitization in Parkinsonian mice. *Neuron* 87 976–988. 10.1016/j.neuron.2015.08.022 26335644 PMC4559856

[B11] Bráz J. M. Sharif-Naeini R. Vogt D. Kriegstein A. Alvarez-Buylla A. Rubenstein J. L. (2012). Forebrain GABAergic neuron precursors integrate into adult spinal cord and reduce injury-induced neuropathic pain. *Neuron* 74 663–675. 10.1016/j.neuron.2012.02.033 22632725 PMC3361692

[B12] Bráz J. M. Wang X. Guan Z. Rubenstein J. L. Basbaum A. I. (2015). Transplant-mediated enhancement of spinal cord GABAergic inhibition reverses paclitaxel-induced mechanical and heat hypersensitivity. *Pain* 156 1084–1091. 10.1097/j.pain.0000000000000152 25760475 PMC4431911

[B13] Buckmaster P. S. Jongen-Rêlo A. L. (1999). Highly specific neuron loss preserves lateral inhibitory circuits in the dentate gyrus of kainate-induced epileptic rats. *J. Neurosci.* 19 9519–9529. 10531454 10.1523/JNEUROSCI.19-21-09519.1999 PMC6782907

[B14] Busche M. A. Eichhoff G. Adelsberger H. Abramowski D. Wiederhold K. H. Haass C. (2008). Clusters of hyperactive neurons near amyloid plaques in a mouse model of Alzheimer’s disease. *Science* 321 1686–1689. 10.1126/science.1162844 18802001

[B15] Butt S. J. Fuccillo M. Nery S. Noctor S. Kriegstein A. Corbin J. G. (2005). The Temporal and spatial origins of cortical interneurons predict their physiological subtype. *Neuron* 48 591–604. 10.1016/j.neuron.2005.09.034 16301176

[B16] Cajal S. R. (1928). Degeneration and regeneration of the nervous system. Oxford: Oxford Press, 799–802. 10.1093/acprof:oso/9780195065169.001.0001

[B17] Calcagnotto M. E. Zipancic I. Piquer-Gil M. Mello L. E. Alvarez-Dolado M. (2010). Grafting of GABAergic precursors rescues deficits in hippocampal inhibition. *Epilepsia* 51(Suppl. 3), 66–70. 10.1111/j.1528-1167.2010.02613.x 20618404

[B18] Capone C. Frigerio S. Fumagalli S. Gelati M. Principato M.-C. Storini C. (2007). Neurosphere-derived cells exert a neuroprotective action by changing the ischemic microenvironment. *PLoS ONE* 2:e373 10.1371/journal.pone.0000373 PMC1847533 17440609

[B19] Caputi A. Rozov A. Blatow M. Monyer H. (2009). Two calretinin-positive gabaergic cell types in layer 2/3 of the mouse neocortex provide different forms of inhibition. *Cereb. Cortex* 19 1345–1359. 10.1093/cercor/bhn175 18842664

[B20] Casarosa S. Fode C. Guillemot F. (1999). Mash1 regulates neurogenesis in the ventral telencephalon. *Development* 126 525–534. 9876181 10.1242/dev.126.3.525

[B21] Cazorla M. de Carvalho F. D. Chohan M. O. Shegda M. Chuhma N. Rayport S. (2014). Dopamine D2 receptors regulate the anatomical and functional balance of basal ganglia circuitry. *Neuron* 81 153–164. 10.1016/j.neuron.2013.10.041 24411738 PMC3899717

[B22] Clark W. E. L. (1940). Neuronal differentiation in implanted fœtal cortical tissue. *J. Neurol. Psychiatry* 3 263–272. 10.1136/jnnp.3.3.263 21610978 PMC1088186

[B23] Cohen I. Navarro V. Clemenceau S. Baulac M. Miles R. (2002). On the origin of interictal activity in human temporal lobe epilepsy in vitro. *Science* 298 1418–1421. 10.1126/science.1076510 12434059

[B24] Colasante G. Lignani G. Rubio A. Medrihan L. Yekhlef L. Sessa A. (2015). Rapid conversion of fibroblasts into functional forebrain GABAergic interneurons by direct genetic reprogramming. *Cell Stem Cell* 17 719–734. 10.1016/j.stem.2015.09.002 26526726

[B25] Cossart R. Dinocourt C. Hirsch J. C. Merchan-Perez A. De Felipe J. Ben-Ari Y. (2001). Dendritic but not somatic GABAergic inhibition is decreased in experimental epilepsy. *Nat. Neurosci.* 4 52–62. 10.1038/82900 11135645

[B26] Cummings B. J. Uchida N. Tamaki S. J. Salazar D. L. Hooshmand M. Summers R. (2005). Human neural stem cells differentiate and promote locomotor recovery in spinal cord-injured mice. *Proc. Natl. Acad. Sci. U.S.A* 102 14069–14074. 10.1073/pnas.0507063102 16172374 PMC1216836

[B27] Cunningham M. Cho J. H. Leung A. Savvidis G. Ahn S. Moon M. (2014). hPSC-derived maturing GABAergic interneurons ameliorate seizures and abnormal behavior in epileptic mice. *Cell Stem Cell* 15 559–573. 10.1016/j.stem.2014.10.006 25517465 PMC4270101

[B28] Davies P. Katzman R. Terry R. D. (1980). Reduced somatostatin-like immunoreactivity in cerebral cortex from cases of Alzheimer disease and Alzheimer senile dementa. *Nature* 288 279–280. 10.1038/288279a0 6107862

[B29] Davis M. F. Figueroa Velez D. X. Guevarra R. P. Yang M. C. Habeeb M. Carathedathu M. C. (2015). Inhibitory neuron transplantation into adult visual cortex creates a new critical period that rescues impaired vision. *Neuron* 86 1055–1066. 10.1016/j.neuron.2015.03.062 25937171 PMC4441572

[B30] De la Cruz E. Zhao M. Guo L. Ma H. Anderson S. A. Schwartz T. (2011). Interneuron progenitors attenuate the power of acute focal ictal discharges. *Neurotherapeutics* 8 763–773. 10.1007/s13311-011-0058-9 21748528 PMC3250298

[B31] de Lanerolle N. C. Kim J. H. Robbins R. J. Spencer D. D. (1989). Hippocampal interneuron loss and plasticity in human temporal lobe epilepsy. *Brain Res.* 495 387–395. 10.1016/0006-8993(89)90234-5 2569920

[B32] DeFelipe J. (1999). Chandelier cells and epilepsy. *Brain* 122(Pt 10), 1807–1822. 10.1093/brain/122.10.1807 10506085

[B33] Doudna J. A. Charpentier E. (2014). The New frontier of genome engineering with CRISPR-Cas9. *Science* 346:1258096 10.1126/science.1258096 25430774

[B34] Drew G. M. Siddall P. J. Duggan A. W. (2004). Mechanical allodynia following contusion injury of the rat spinal cord is associated with loss of GABAergic inhibition in the dorsal horn. *Pain* 109 379–388. 10.1016/j.pain.2004.02.007 15157699

[B35] Dunn E. H. (1917). Primary and secondary findings in a series of attempts to transplant cerebral cortex in the albino rat. *J. Comp. Neurol.* 27 565–582. 10.1002/cne.900270403

[B36] Fagiolini M. Hensch T. K. (2000). Inhibitory threshold for critical-period activation in primary visual cortex. *Nature* 404 183–186. 10.1038/35004582 10724170

[B37] Flames N. Long J. E. Garratt A. N. Fischer T. M. Gassmann M. Birchmeier C. (2004). Short- and Long-range attraction of cortical GABAergic interneurons by neuregulin-1. *Neuron* 44 251–261. 10.1016/j.neuron.2004.09.028 15473965

[B38] Fuentealba L. C. Rompani S. B. Parraguez J. I. Obernier K. Romero R. Cepko C. L. (2015). Embryonic origin of postnatal neural stem cells. *Cell* 161 1644–1655. 10.1016/j.cell.2015.05.041 26091041 PMC4475276

[B39] Gage F. H. Björklund A. Stenevi U. Dunnett S. B. Kelly P. A. (1984). Intrahippocampal septal grafts ameliorate learning impairments in aged rats. *Science* 225 533–536. 10.1126/science.6539949 6539949

[B40] Gage N. H. Dunnett S. B. Stenevi U. Björklund A. (1983). Aged rats: recovery of motor impairments by intrastriatal nigral grafts. *Science* 221 966–969. 10.1126/science.6879196 6879196

[B41] Gelman D. Griveau A. Dehorter N. Teissier A. Varela C. Pla R. (2011). A wide diversity of cortical GABAergic interneurons derives from the embryonic preoptic area. *J. Neurosci* 31 16570–16580. 10.1523/JNEUROSCI.4068-11.2011 22090484 PMC6633309

[B42] Gilani A. I. Chohan M. O. Inan M. Schobel S. A. Chaudhury N. H. Paskewitz S. (2014). Interneuron precursor transplants in adult hippocampus reverse psychosis-relevant features in a mouse model of hippocampal disinhibition. *Proc. Natl. Acad. Sci. U.S.A.* 111 7450–7455. 10.1073/pnas.1316488111 24794528 PMC4034251

[B43] Gittis A. H. Hang G. B. LaDow E. S. Shoenfeld L. R. Atallah B. V. Finkbeiner S. (2011). Rapid target-specific remodeling of fast-spiking inhibitory circuits after loss of dopamine. *Neuron* 71 858–868. 10.1016/j.neuron.2011.06.035 21903079 PMC3170520

[B44] Glickstein S. B. Moore H. Slowinska B. Racchumi J. Suh M. Chuhma N. (2007). Selective cortical interneuron and GABA deficits in cyclin D2-Null mice. *Development* 134 4083–4093. 10.1242/dev.008524 17965053 PMC3396210

[B45] Goldberg N. R. S. Caesar J. Park A. Sedgh S. Finogenov G. Masliah E. (2015). Neural stem cells rescue cognitive and motor dysfunction in a transgenic model of dementia with lewy bodies through a BDNF-dependent mechanism. *Stem Cell Rep.* 5 791–804. 10.1016/j.stemcr.2015.09.008 PMC4649255 26489892

[B46] Grouselle D. Winsky-Sommerer R. David J. P. Delacourte A. Dournaud P. Epelbaum J. (1998). Loss of somatostatin-like immunoreactivity in the frontal cortex of Alzheimer patients carrying the apolipoprotein epsilon 4 allele. *Neurosci. Lett.* 255 21–24. 10.1016/S0304-3940(98)00698-3 9839717

[B47] Gulyás A. I. Hájos N. Freund T. F. (1996). Interneurons containing calretinin are specialized to control other interneurons in the rat hippocampus. *J. Neurosci.* 16 3397–3411. 8627375 10.1523/JNEUROSCI.16-10-03397.1996 PMC6579144

[B48] Gutin G. Fernandes M. Palazzolo L. Paek H. Yu K. Ornitz D. M. (2006). FGF signalling generates ventral telencephalic cells independently of SHH. *Development* 133 2937–2946. 10.1242/dev.02465 16818446

[B49] Hammad M. Schmidt S. L. Zhang X. Bray R. Frohlich F. Ghashghaei H. T. (2015). Transplantation of GABAergic interneurons into the neonatal primary visual cortex reduces absence seizures in stargazer mice. *Cereb. Cortex* 25 2970–2979. 10.1093/cercor/bhu094 24812085 PMC4537440

[B50] Harrison P. J. (2015). GABA circuitry, cells and molecular regulation in schizophrenia: life in the graveyard. *Schizophr. Res.* 167 108–110. 10.1016/j.schres.2015.02.003 25694185

[B51] Hashimoto T. Bazmi H. H. Mirnics K. Wu Q. Sampson A. R. Lewis D. A. (2008). Conserved regional patterns of GABA-related transcript expression in the neocortex of subjects with schizophrenia. *Am. J. Psychiatry* 165 479–489. 10.1176/appi.ajp.2007.07081223 18281411 PMC2894608

[B52] Hashimoto T. Volk D. W. Eggan S. M. Mirnics K. Pierri J. N. Sun Z. (2003). Gene expression deficits in a subclass of GABA neurons in the prefrontal cortex of subjects with schizophrenia. *J. Neurosci* 23 6315–6326. 10.2967/jnumed.108.060368 12867516 PMC6740534

[B53] Hattiangady B. Shetty A. K. (2012). Neural stem cell grafting counteracts hippocampal injury-mediated impairments in mood, memory, and neurogenesis. *Stem Cells Transl. Med.* 1 696–708. 10.5966/sctm.2012-0050 23197876 PMC3612501

[B54] Henderson K. W. Gupta J. Tagliatela S. Litvina E. Zheng X. T. Van Zandt M. A. (2014). Long-term seizure suppression and optogenetic analyses of synaptic connectivity in epileptic mice with hippocampal grafts of GABAergic interneurons. *J. Neurosci.* 34 13492–13504. 10.1523/JNEUROSCI.0005-14.2014 25274826 PMC4180479

[B55] Hensch T. K. (2005). Critical period plasticity in local cortical circuits. *Nat. Rev. Neurosci.* 6 877–888. 10.1038/nrn1787 16261181

[B56] Howard M. A. Rubenstein J. L. Baraban S. C. (2014). Bidirectional homeostatic plasticity induced by interneuron cell death and transplantation in vivo. *Proc. Natl. Acad. Sci. U.S.A.* 111 492–497. 10.1073/pnas.1307784111 24344303 PMC3890856

[B57] Hunt R. F. Baraban S. C. (2015). Interneuron transplantation as a treatment for epilepsy. *Cold Spring Harb. Perspect. Med.* 5:a022376 10.1101/cshperspect.a022376 PMC4665034 26627452

[B58] Hunt R. F. Girskis K. M. Rubenstein J. L. Alvarez-Buylla A. Baraban S. C. (2013). GABA progenitors grafted into the adult epileptic brain control seizures and abnormal behavior. *Nat. Neurosci.* 16 692–697. 10.1038/nn.3392 23644485 PMC3665733

[B59] Jaiswal M. K. Keros S. Zhao M. Inan M. Schwartz T. H. Anderson S. A. (2015). Reduction in focal ictal activity following transplantation of MGE interneurons requires expression of the GABAA receptor α4 subunit. *Front. Cell. Neurosci.* 9:127 10.3389/fncel.2015.00127 PMC4391265 25914623

[B60] Jakeman L. B. Reier P. J. (1991). Axonal projections between fetal spinal cord transplants and the adult rat spinal cord: a neuroanatomical tracing study of local interactions. *J. Comp. Neurol.* 307 311–334. 10.1002/cne.903070211. 1713233

[B61] Klausberger T. Magill P. J. Márton L. F. Roberts J. D. Cobden P. M. Buzsáki G. (2003). Brain-state- and cell-type-specific firing of hippocampal interneurons in Vivo. *Nature* 421 844–848. 12594513 10.1038/nature01374

[B62] Klausberger T. Somogyi P. (2008). Neuronal diversity and temporal dynamics: the unity of hippocampal circuit operations. *Science* 321 53–57. 10.1126/science.1149381 18599766 PMC4487503

[B63] Kobayashi M. Buckmaster P. S. (2003). Reduced inhibition of dentate granule cells in a model of temporal Lobe Epilepsy. *J. Neurosci.* 23 2440–2452. 12657704 10.1523/JNEUROSCI.23-06-02440.2003 PMC6741996

[B64] Konradi C. Yang C. K. Zimmerman E. I. Lohmann K. M. Gresch P. Pantazopoulos H. (2011). Hippocampal interneurons are abnormal in schizophrenia. *Schizophr. Res.* 131 165–173. 10.1016/j.schres.2011.06.007 21745723 PMC3159834

[B65] Kromer L. F. Björklund A. Stenevi U. (1983). Intracephalic embryonic neural implants in the adult rat brain. I. Growth and mature organization of brainstem, cerebellar, and hippocampal implants. *J. Comp. Neurol.* 218 433–459. 10.1002/cne.902180408 6619323

[B66] Lee J. P. Jeyakumar M. Gonzalez R. Takahashi H. Lee P. J. Baek R. C. (2007). Stem cells act through multiple mechanisms to benefit mice with neurodegenerative metabolic disease. *Nat. Med.* 13 439–447. 10.1038/nm1548 17351625

[B67] Lewis D. A. Curley A. A. Glausier J. R. Volk D. W. (2012). Cortical parvalbumin interneurons and cognitive dysfunction in schizophrenia. *Trends Neurosci.* 35 57–67. 10.1016/j.tins.2011.10.004 22154068 PMC3253230

[B68] Li Y. Raisman G. (1993). Long axon growth from embryonic neurons transplanted into myelinated tracts of the adult rat spinal cord. *Brain Res.* 629 115–127. 10.1016/0006-8993(93)90489-A 8287266

[B69] Liang P. Liu J. Xiong J. Liu Q. Zhao J. Liang H. (2014). Neural stem cell-conditioned medium protects neurons and promotes propriospinal neurons relay neural circuit reconnection after spinal cord injury. *Cell Transplant.* 23(Suppl. 1), S45–S56. 10.3727/096368914X684989 25333841

[B70] Liodis P. Denaxa M. Grigoriou M. Akufo-Addo C. Yanagawa Y. Pachnis V. (2007). Lhx6 activity is required for the normal migration and specification of cortical interneuron subtypes. *J. Neurosci.* 27 3078–3089. 10.1523/JNEUROSCI.3055-06.2007 17376969 PMC6672459

[B71] Liu Y. Weick J. P. Liu H. Krencik R. Zhang X. Ma L. (2013). Medial ganglionic eminence-like cells derived from human embryonic stem cells correct learning and memory deficits. *Nat. Biotechnol.* 31 440–447. 10.1038/nbt.2565 23604284 PMC3711863

[B72] Low W. C. Lewis P. R. Bunch S. T. Dunnett S. B. Thomas S. R. Iversen S. D. (1982). Function recovery following neural transplantation of embryonic septal nuclei in adult rats with septohippocampal lesions. *Nature* 300 260–262. 10.1038/300260a0 7144881

[B73] Lu P. Wang Y. Graham L. McHale K. Gao M. Wu D. (2012). Long-distance growth and connectivity of neural stem cells after severe spinal cord injury. *Cell* 150 1264–1273. 10.1016/j.cell.2012.08.020 22980985 PMC3445432

[B74] Lund R. D. Hauschka S. D. (1976). Transplanted neural tissue develops connections with host rat brain. *Science* 193 582–584. 959815 10.1126/science.959815

[B75] Mallet N. Ballion B. Le Moine C. Gonon F. (2006). Cortical inputs and GABA interneurons imbalance projection neurons in the striatum of parkinsonian rats. *J Neurosci.* 26 3875–3884. 10.1523/JNEUROSCI.4439-05.2006 16597742 PMC6674115

[B76] Marín O. Yaron A. Bagri A. Tessier-Lavigne M. Rubenstein J. L. (2001). Sorting of striatal and cortical interneurons regulated by semaphorin-neuropilin interactions. *Science* 293 872–875. 10.1126/science.1061891 11486090

[B77] Maroof A. M. Keros S. Tyson J. A. Ying S. W. Ganat Y. M. Merkle F. T. (2013). Directed differentiation and functional maturation of cortical interneurons from human embryonic stem cells. *Cell Stem Cell* 12 559–572. 10.1016/j.stem.2013.04.008 23642365 PMC3681523

[B78] Martínez-Cerdeño V. Noctor S. C. Espinosa A. Ariza J. Parker P. Orasji S. (2010). Embryonic MGE precursor cells grafted into adult rat striatum integrate and ameliorate motor symptoms in 6-OHDA-lesioned rats. *Cell Stem Cell* 6 238–250. 10.1016/j.stem.2010.01.004 20207227 PMC4075336

[B79] Mathern G. W. Babb T. L. Pretorius J. K. Leite J. P. (1995). Reactive synaptogenesis and neuron densities for neuropeptide, Y., somatostatin, and glutamate decarboxylase immunoreactivity in the epileptogenic human fascia dentata. *J. Neurosci.* 15(5 Pt 2), 3990–4004. 7751960 10.1523/JNEUROSCI.15-05-03990.1995 PMC6578224

[B80] Merkle F. T. Alvarez-Buylla A. (2006). Neural stem cells in mammalian development. *Curr. Opin. Cell Biol.* 18 704–709. 10.1016/j.ceb.2006.09.008 17046226

[B81] Miyoshi G. Hjerling-Leffler J. Karayannis T. Sousa V. H. Butt S. J. B. Battiste J. (2010). Genetic fate mapping reveals that the caudal ganglionic eminence produces a large and diverse population of superficial cortical interneurons. *J. Neurosci.* 30 1582–1594. 10.1523/JNEUROSCI.4515-09.2010 20130169 PMC2826846

[B82] Möhler H. Fritschy J.-M. Crestani F. Hensch T. Rudolph U. (2004). Specific GABA(A) circuits in brain development and therapy. *Biochem.* *Pharmacol.* 68 1685–1690. 10.1016/j.bcp.2004.07.025 15451412

[B83] Moore K. A. Kohno T. Karchewski L. A. Scholz J. Baba H. Woolf C. J. (2002). Partial peripheral nerve injury promotes a selective loss of GABAergic inhibition in the superficial dorsal horn of the spinal cord. *J. Neurosci.* 22 6724–6731. 12151551 10.1523/JNEUROSCI.22-15-06724.2002 PMC6758148

[B84] Morris H. M. Hashimoto T. Lewis D. A. (2008). Alterations in somatostatin mRNA expression in the dorsolateral prefrontal cortex of subjects with schizophrenia or schizoaffective disorder. *Cerebral Cortex* 18 1575–1587. 10.1093/cercor/bhm186 18203698 PMC2888087

[B85] Munro G. Ahring P. K. Mirza N. R. (2009). Developing analgesics by enhancing spinal inhibition after injury: GABAA receptor subtypes as novel targets. *Trends Pharmacol. Sci.* 30 453–459. 10.1016/j.tips.2009.06.004 19729210

[B86] Muotri A. R. Gage F. H. (2006). Generation of neuronal variability and complexity. *Nature* 441 1087–1093. 10.1038/nature04959 16810244

[B87] Murray A. J. Sauer J. F. Riedel G. McClure C. Ansel L. Cheyne L. (2011). Parvalbumin-positive CA1 interneurons are required for spatial working but not for reference memory. *Nat. Neurosci.* 14 297–299. 10.1038/nn.2751 21278730 PMC3064406

[B88] Nery S. Fishell G. Corbin J. G. (2002). The caudal ganglionic eminence is a source of distinct cortical and subcortical cell populations. *Nat. Neurosci.* 5 1279–1287. 10.1038/nn971 12411960

[B89] Nornes H. Bjorklund A. Stenevi U. (1983). Reinnervation of the denervated adult spinal cord of rats by intraspinal transplants of embryonic brain stem neurons. *Cell Tissue Res.* 230 15–35. 10.1007/BF00216024 6850760

[B90] Ogiwara I. Miyamoto H. Morita N. Atapour N. Mazaki E. Inoue I. (2007). Nav1.1 localizes to axons of parvalbumin-positive inhibitory interneurons: a circuit basis for epileptic seizures in mice carrying an scn1a gene mutation. *J. Neurosci.* 27 5903–5914. 10.1523/JNEUROSCI.5270-06.2007 17537961 PMC6672241

[B91] Ourednik J. Ourednik V. Lynch W. P. Schachner M. Snyder E. Y. (2002). Neural stem cells display an inherent mechanism for rescuing dysfunctional neurons. *Nat. Biotechnol.* 20 1103–1110. 10.1038/nbt750 12379867

[B92] Palop J. J. Chin J. Roberson E. D. Wang J. Thwin M. T. Bien-Ly N. (2007). Aberrant excitatory neuronal activity and compensatory remodeling of inhibitory hippocampal circuits in mouse models of alzheimer’s disease. *Neuron* 55 697–711. 10.1016/j.neuron.2007.07.025 17785178 PMC8055171

[B93] Panuccio G. Curia G. Colosimo A. Cruccu G. Avoli M. (2009). Epileptiform synchronization in the cingulate cortex. *Epilepsia* 50 521–536. 10.1111/j.1528-1167.2008.01779.x 19178556 PMC4879611

[B94] Park K. I. Teng Y. D. Snyder E. Y. (2002). The injured brain interacts reciprocally with neural stem cells supported by scaffolds to reconstitute lost tissue. *Nat. Biotechnol.* 20 1111–1117. 10.1038/nbt751 12379868

[B95] Paulsen O. Moser E. I. (1998). A model of hippocampal memory encoding and retrieval: GABAergic control of synaptic plasticity. *Trends Neurosci.* 21 273–278. 10.1016/S0166-2236(97)01205-8 9683315

[B96] Peñagarikano O. Abrahams B. S. Herman E. I. Winden K. D. Gdalyahu A. Dong H. (2011). Absence of CNTNAP2 leads to epilepsy, neuronal migration abnormalities, and core autism-related deficits. *Cell* 147 235–246. 10.1016/j.cell.2011.08.040 21962519 PMC3390029

[B97] Petros T. J. Bultje R. S. Ross M. E. Fishell G. Anderson S. A. (2015). Apical versus basal neurogenesis directs cortical interneuron subclass fate. *Cell Rep.* 13 1090–1095. 10.1016/j.celrep.2015.09.079 26526999 PMC4704102

[B98] Redmond DE Jr Bjugstad K. B. Teng Y. D. Ourednik V. Ourednik J. Wakeman D. R. (2007). Behavioral improvement in a primate parkinson’s model is associated with multiple homeostatic effects of human neural stem cells. *Proc.* *Natl. Acad. Sci. U. S.A.* 104 12175–12180. 10.1073/pnas.0704091104 PMC1896134 17586681

[B99] Salin P. López I. P. Kachidian P. Barroso-Chinea P. Rico A. J. Gómez-Bautista V. (2009). Changes to interneuron-driven striatal microcircuits in a rat model of parkinson’s disease. *Neurobiol. Dis.* 34 545–552. 10.1016/j.nbd.2009.03.006 19341798

[B100] Schobel S. A. Chaudhury N. H. Khan U. A. Paniagua B. Styner M. A. Asllani I. (2013). Imaging patients with psychosis and a mouse model establishes a spreading pattern of hippocampal dysfunction and implicates glutamate as a driver. *Neuron* 78 81–93. 10.1016/j.neuron.2013.02.011 23583108 PMC3966570

[B101] Shen W. Flajolet M. Greengard P. Surmeier D. J. (2008). Dichotomous dopaminergic control of striatal synaptic plasticity. *Science* 321 848–851. 10.1126/science.1160575 18687967 PMC2833421

[B102] Shyh-Chang N. Daley G. Q. Cantley L. C. (2013). Stem cell metabolism in tissue development and aging. *Development* 140 2535–2547. 10.1242/dev.091777 23715547 PMC3666381

[B103] Sloviter R. S. (1987). Decreased hippocampal inhibition and a selective loss of interneurons in experimental epilepsy. *Science* 235 73–76. 10.1126/science.2879352 2879352

[B104] Sohal V. S. Zhang F. Yizhar O. Deisseroth K. (2009). Parvalbumin neurons and gamma rhythms enhance cortical circuit performance. *Nature* 459 698–702. 10.1038/nature07991 19396159 PMC3969859

[B105] Southwell D. G. Froemke R. C. Alvarez-Buylla A. Stryker M. P. Gandhi S. P. (2010). Cortical plasticity induced by inhibitory neuron transplantation. *Science* 327 1145–1148. 10.1126/science.1183962 20185728 PMC3164148

[B106] Southwell D. G. Nicholas C. R. Basbaum A. I. Stryker M. P. Kriegstein A. R. Rubenstein J. L. (2014). Interneurons from embryonic development to cell-based therapy. *Science* 344:1240622 10.1126/science.1240622 PMC4056344 24723614

[B107] Sundstrom L. E. Brana C. Gatherer M. Mepham J. Rougier A. (2001). Somatostatin- and neuropeptide Y-synthesizing neurones in the fascia dentata of humans with temporal lobe epilepsy. *Brain* 124 688–697. 10.1093/brain/124.4.688 11287369

[B108] Sussel L. Marin O. Kimura S. Rubenstein J. L. (1999). Loss of Nkx2.1 homeobox gene function results in a ventral to dorsal molecular respecification within the basal telencephalon: evidence for a transformation of the pallidum into the striatum. *Development* 126 3359–3370. 10393115 10.1242/dev.126.15.3359

[B109] Tanaka D. H. Toriumi K. Kubo K. Nabeshima T. Nakajima K. (2011). GABAergic precursor transplantation into the prefrontal cortex prevents phencyclidine-induced cognitive deficits. *J. Neurosci.* 31 14116–14125. 10.1523/JNEUROSCI.2786-11.2011 21976496 PMC6623672

[B110] Tang Y. Stryker M. P. Alvarez-Buylla A. Espinosa J. S. (2014). Cortical plasticity induced by transplantation of embryonic somatostatin or parvalbumin interneurons. *Proc.* *Natl. Acad. Sci. U.S.A.* 111 18339–18344. 10.1073/pnas.1421844112 PMC4280644 25489113

[B111] Tepper J. M. Bolam J. P. (2004). Functional diversity and specificity of neostriatal interneurons. *Curr.* *Opin. Neurobiol.* 14 685–692. 10.1016/j.conb.2004.10.003 15582369

[B112] Tidd C. W. (1932). The transplantation of spinal ganglia in the white rat. A study of the morphological changes in surviving cells. *J. Comp. Neurol.* 55 531–543. 10.1002/cne.900550213

[B113] Tong L. M. Djukic B. Arnold C. Gillespie A. K. Yoon S. Y. Wang M. M. (2014). Inhibitory interneuron progenitor transplantation restores normal learning and memory in ApoE4 knock-in mice without or with Aβ accumulation. *J. Neurosci.* 34 9506–9515. 10.1523/JNEUROSCI.0693-14.2014 25031394 PMC4099537

[B114] Tricoire L. Pelkey K. A. Erkkila B. E. Jeffries B. W. Yuan X. McBain C. J. (2011). A blueprint for the spatiotemporal origins of mouse hippocampal interneuron diversity. *J. Neurosci.* 31 10948–10970. 10.1523/JNEUROSCI.0323-11.2011 21795545 PMC3163481

[B115] Tyson J. A. Goldberg E. M. Maroof A. M. Xu Q. Petros T. J. Anderson S. A. (2015). Duration of culture and sonic hedgehog signaling differentially specify PV versus SST cortical interneuron fates from embryonic stem cells. *Development* 142 1267–1278. 10.1242/dev.111526 25804737 PMC4378243

[B116] Verret L. Mann E. O. Hang G. B. Barth A. M. Cobos I. Ho K. (2012). Inhibitory interneuron deficit links altered network activity and cognitive dysfunction in alzheimer model. *Cell* 149 708–721. 10.1016/j.cell.2012.02.046 22541439 PMC3375906

[B117] Waldau B. Hattiangady B. Kuruba R. Shetty A. K. (2010). Medial ganglionic eminence-derived neural stem cell grafts ease spontaneous seizures and restore GDNF expression in a rat model of chronic temporal lobe epilepsy. *Stem Cells* 28 1153–1164. 10.1002/stem.446 20506409 PMC2933789

[B118] Wall N. R. De La Parra M. Callaway E. M. Kreitzer A. C. (2013). Differential innervation of direct- and indirect-pathway striatal projection neurons. *Neuron* 79 347–360. 10.1016/j.neuron.2013.05.014 23810541 PMC3729794

[B119] Walsh C. Cepko C. (1988). Clonally related cortical cells show several migration patterns. *Science* 241 1342–1345. 10.1126/science.3137660 3137660

[B120] Walsh C. Cepko C. L. (1993). Clonal dispersion in proliferative layers of developing cerebral cortex. *Nature* 362 632–635. 10.1038/362632a0 8464513

[B121] Weerakkody T. N. Patel T. P. Yue C. Takano H. Anderson H. C. Meaney D. F. (2013). Engraftment of nonintegrating neural stem cells differentially perturbs cortical activity in a dose-dependent manner. *Mol. Therapy* 21 2258–2267. 10.1038/mt.2013.163 PMC3863790 23831593

[B122] Willis R. A. (1935). Experiments on the intracerebral implantation of embryo tissues in rats. *Proc. R. Soc. Lond. B. Biol. Sci.* 117 400–412. 10.1098/rspb.1935.0036

[B123] Wittner L. Eross L. Czirják S. Halász P. Freund T. F. Maglóczky Z. (2005). Surviving CA1 pyramidal cells receive intact perisomatic inhibitory input in the human epileptic hippocampus. *Brain* 128 138–152. 10.1093/brain/awh339 15548550

[B124] Wittner L. Eross L. Szabó Z. Tóth S. Czirják S. Halász P. (2002). Synaptic reorganization of calbindin-positive neurons in the human hippocampal CA1 region in temporal lobe epilepsy. *Neuroscience* 115 961–978. 10.1016/S0306-4522(02)00264-6 12435433

[B125] Wittner L. Maglóczky Z. Borhegyi Z. Halász P. Tóth S. Eross L. (2001). Preservation of perisomatic inhibitory input of granule cells in the epileptic human dentate gyrus. *Neuroscience* 108 587–600. 10.1016/S0306-4522(01)00446-8 11738496

[B126] Wonders C. P. Anderson S. A. (2006). The origin and specification of cortical interneurons. *Nat. Rev. Neurosci.* 7 687–696. 10.1038/nrn1954 16883309

[B127] Xu Q. Cobos I. De La Cruz E. Rubenstein J. L. Anderson S. A. (2004). Origins of cortical interneuron subtypes. *J. Neurosci.* 24 2612–2622. 10.1523/JNEUROSCI.5667-03.2004 15028753 PMC6729522

[B128] Xu Q. Guo L. Moore H. Waclaw R. R. Campbell K. Anderson S. A. (2010). Sonic hedgehog signaling confers ventral telencephalic progenitors with distinct cortical interneuron fates. *Neuron* 65 328–340. 10.1016/j.neuron.2010.01.004 20159447 PMC2868511

